# Growth Rate and Not Growing Season Explains the Increased Productivity of Masson Pine in Mixed Stands

**DOI:** 10.3390/plants14030313

**Published:** 2025-01-21

**Authors:** Chunmei Bai, Wendi Zhao, Marcin Klisz, Sergio Rossi, Weijun Shen, Xiali Guo

**Affiliations:** 1 Guangxi Key Laboratory of Forest Ecology and Conservation, State Key Laboratory for Conservation and Utilization of Agro-Bioresources, College of Forestry, Guangxi University, Nanning 530004, China; sdycre@163.com (C.B.); zwendi1012@163.com (W.Z.); 2 Dendrolab IBL, Department of Silviculture and Genetics, Forest Research Institute, 05-090 Raszyn, Poland; m.klisz@ibles.waw.pl; 3 Laboratoire sur les Ecosystèmes Terrestres Boréaux, Département des Sciences Fondamentales, Université du Québec à Chicoutimi, Chicoutimi, QC G7H 2B1, Canada; srossi@uqac.ca

**Keywords:** subtropical forest, overyielding, mixing effect, unevenly aged stands, xylem formation, *Pinus massoniana*

## Abstract

Increased tree species diversity can promote forest production by reducing intra-specific competition and promoting an efficient unitization of resources. However, questions remain on whether and how mixed stands affect the dynamics of intra–annual xylem formation in trees, especially in subtropical forests. In this study, we randomly selected 18 trees from a monoculture of 63-year-old Masson pine (*Pinus massoniana*) growing in pure stands and mixed them with 39-year-old *Castanopsis hystrix* in Pinxiang, southern China. A total of 828 microcores were collected biweekly throughout the growing season from 2022 to 2023 to monitor the intra-annual xylem formation. Cell production started in early March and ended in late December and lasted about 281 to 284 days. Xylem phenology was similar between mixed and pure stands. During both seasons, the Masson pine in mixed stands showed higher xylem production and growth rates than those in pure stands. The Masson pine in mixed stands produced 45–51 cells in 2022 (growth rate of 0.22 cells day^−1^) and 35–41 cells in 2023 (0.17 cells day^−1^). Growth rate, and not growth seasons, determined the superior xylem growth in the mixed stands. Our study shows that after 39 years of management, Masson pine and *C. hystrix* unevenly aged mixed stands have a significant positive mixing effect on Masson pine xylem cell production, which demonstrates that monitoring intra-annual xylem growth dynamics can be an important tool to evaluate the effect of species composition and reveal the mechanisms to promote tree growth behind the mixing effect.

## 1. Introduction

Monospecific stands have been preferred for plantations for simple management and economic benefits [1]. However, the simple structure and function of pure stands can lead to the disruption of hydrological cycles, soil erosion and degradation, fire risk, and susceptibility to windstorms, as well as the decreased biodiversity, productivity, and resilience of forest ecosystems [2]. The transformation of monospecific stands into mixed stands is key to mitigating these risks. The establishment of coniferous and broad-leafed unevenly aged mixed stands can fully utilize the self-development, self-sustainment, and self-regulation capabilities of forests, thereby realizing the multi-functional effects of plantations [3,4].

Previous studies have demonstrated that increased tree species diversity could promote productivity, and thus transforming pure stands into mixed stands may have a significant effect on yield [5]. This study showed that height, diameter, and aboveground biomass in mixed stands were higher than those measured in pure stands [6]. One possible explanation for the positive relationship between diversity and productivity is that the inter-species competition in mixed stands is lower, and thus the reduced competition contributes to higher productivity [7,8]. In addition, mixed stands have more complex structures and interspecific interactions, and niche complementarity could promote more efficient allocation of resources (e.g., light, water, and soil nutrients), which in turn contribute to biomass accumulation [9]. As different tree species are limited by different ecological factors, the combination of tree species with different ecological characteristics could increase the ecosystem’s resistance against climatic extremes [10], thereby reducing the negative effects of aboveground net primary productivity due to climatic change [3]. For example, the secondary growth of *Pinus pinea* in mixed stands is enhanced compared to those in mono-specific stands under water stress, indicating that mixed coniferous and deciduous species can effectively buffer the effects of climate change on the stands of the Spanish Northern Plateau [7].

Cambium is the source of wood formation, and it goes dormant to avoid the harsh winter and becomes active in favorable seasons to maximize the availability of resources [11]. In early spring, as the forcing temperature reaches a critical threshold during the ecodormancy phase [12], cambium begins differentiating, ultimately completing the annual xylem growth through cell enlargement, cell wall thickening, and cell maturation [13]. The growing season length and the growth rate of xylem are proven to be the most important factors deciding the xylem production of trees [14]. Under global warming, rising temperature has advanced the onset of xylem formation, thus prolonging the growth season length and increasing xylem cell production [15,16]. However, water stress caused by high temperatures can also reduce the growth rate of xylem during the growing season and decrease xylem cell production [17]. Xylem formation is strongly influenced by various environmental factors, such as temperature [15,18], precipitation, and nutrient supply [19,20,21]. It has been widely recognized that species diversity promotes tree growth [22,23], and mixing effects can indirectly affect wood formation by enhancing the stand microenvironment, such as improving light intensity, soil nutrient content, and moisture, as well as increasing resource utilization efficiency, which ultimately boosts wood production [24,25]. Yet, most previous studies on biodiversity productivity have focused on analyzing basal area increment (BAI) [26,27], and the mechanism of the mixing effect on xylem growth (e.g., by altering the critical phenology of xylem formation (onset, end, growing season length) or growth rate) has not been clarified. Therefore, continuous sampling during the growing season of trees with the micro-core method to obtain high-temporal-resolution xylem sections can accurately capture the key phenological period of xylem growth and quantify cell production and growth rate [28], providing a new idea for evaluating the mixing effects on xylem growth.

Masson pine (*Pinus massoniana* Lamb.), a native species in subtropical China, is widely distributed and has economic importance in Southern Asia [29]. However, more than 90% of Masson pine has been managed in monoculture plantations [30], which is often associated with being highly vulnerable to diseases and insect pests such as nematodes, beetles, and root diseases. Previous studies showed that mixed forests are generally less vulnerable to fire [31] and less sensitive to pest outbreaks and herbivory issues [32]. Unevenly aged mixed plantations of coniferous and broad-leafed trees can increase stand production [33,34]. However, the critical processes by which the mixing effect influences tree radial growth remain unclear. Thus, we selected Masson pine in both pure and mixed stands to test the differences in intra-annual xylem growth dynamics under different treatments. We tested the hypotheses that (i) Masson pine in mixed stands reaches higher wood production compared with those in pure stands, and that (ii) higher wood production is due to an enhanced xylem growth rate.

## 2. Results

### 2.1. Cambial Activity and Xylem Formation Dynamics Between Pure and Mixed Stands

**Figure 1:**
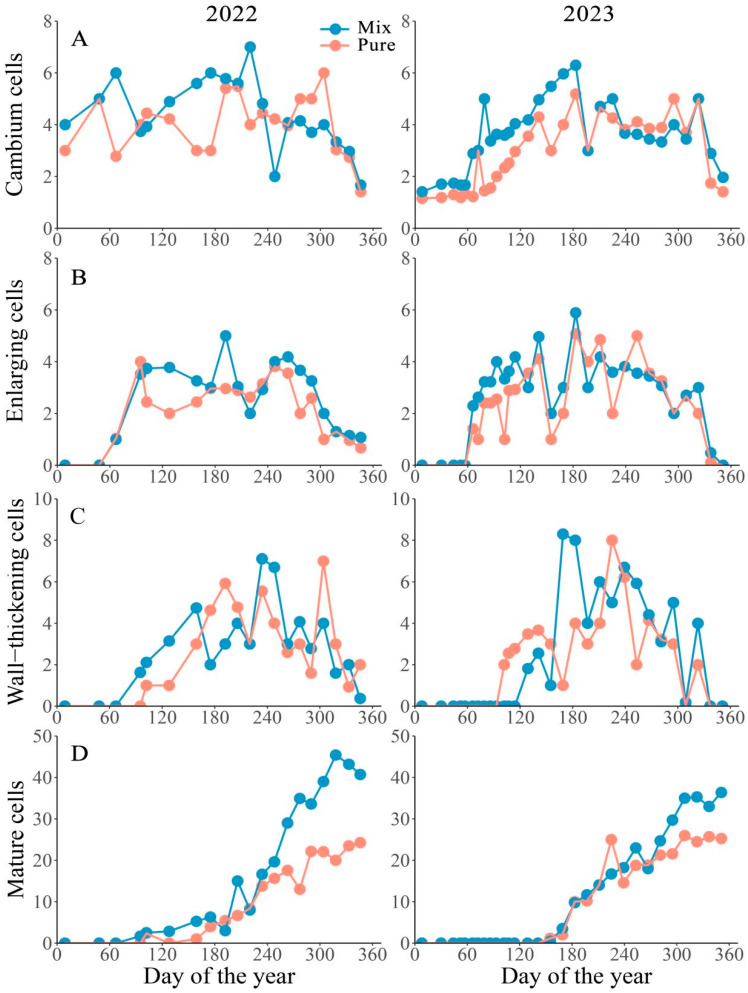
Seasonal dynamics of xylem cell production in different phenological phases, including cambium cells (**A**), enlarging cells (**B**), wall-thickening cells (**C**), and mature cells (**D**), in 2022 and 2023. Mix and Pure represent Masson pine in mixed and pure stands.

The cambial cells and differentiating xylem cells in both pure and mixed stands demonstrated similar growth patterns during 2022 and 2023 (Figure 1). Between January and February, one to three cambial cells remained inactive, with division commencing in March. The number of cambial cells fluctuated between five and seven from DOY 155 to DOY 220, then gradually decreased until December (Figure 1A). Enlarging cells first emerged in March in both stands, with their numbers fluctuating between three and six from April to October (Figure 1B). Two weeks later, the first wall-thickening cell appeared, with the number slightly varying between two to three from April to June (DOY 80–155). Subsequently, it increased sharply to eight before gradually diminishing until December (Figure 1C). The mature cells increased rapidly from DOY 93 to DOY 255 and then varied greatly until the late growth season in December (Figure 1D).

**Figure 2:**
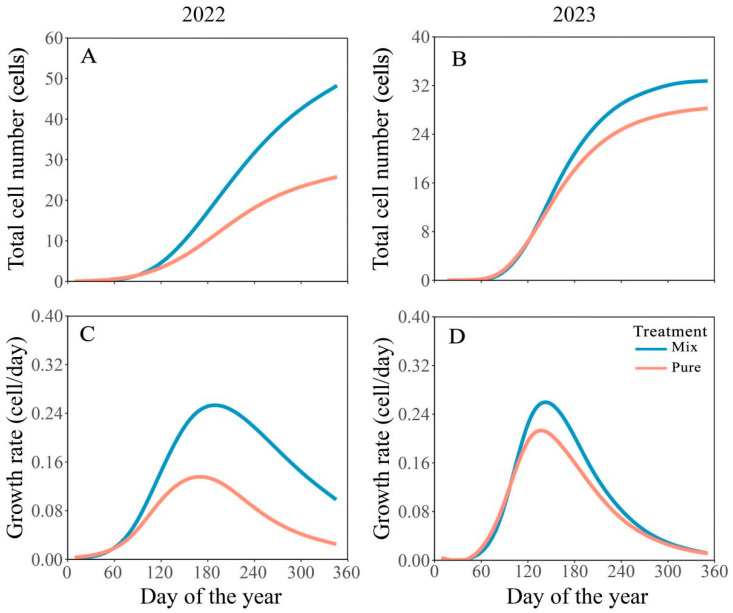
Gompertz function-based predictions of total cell number (**A**,**B**) and growth rate (**C**,**D**) of Masson pine in pure and mixed stands in 2022 and 2023.

The xylem growth of Masson pine showed similar growth patterns in the two years, regardless of whether it was in pure or mixed stands. The growth trend of xylem followed an S-shape (Figure 2A,B), and the growth rate curve was basically consistent with an inverted bell shape (Figure 2C,D). In the mixed stands, the xylem cell production grew slowly during the initial phase of the growing season, from February until the end of May. From June to November, 73% of the total xylem wood formation occurred. Similarly, in the pure stands, the total cell number grew slowly to eight from January to June, and 60% of xylem wood formation was completed from June to November. During both years, the peak growth rate in mixed stands occurred later than in pure stands. In 2022, the time of the maximum growth rate was estimated on DOY 168 ± 8 (pure stands) and 188 ± 5 (mixed stands), respectively. In 2023, the time of the maximum growth rate occurred on DOY 141 ± 4 in pure stands and 155 ± 3 in mixed stands (Table 1).

### 2.2. Comparison of Simulated Parameters of Xylem Formation Between Pure and Mixed Stands

**Figure 3:**
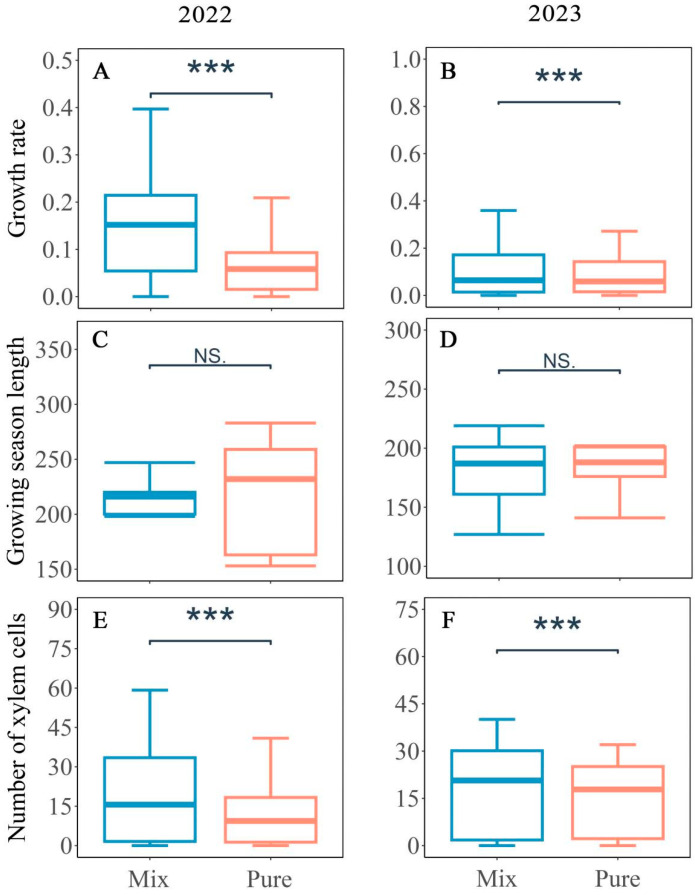
Differences in the growth rate (**A**,**B**), growing season length (**C**,**D**), and the number of xylem cells (**E**,**F**) of Masson pine between pure and mixed stands in 2022 and 2023. Significance was displayed in the figure (***, *p* < 0.001, NS, no significance).

There was no significant difference in the onset, end, and growing season length of the xylem formation between the pure and mixed stands in 2022 and 2023 (Figure 3, *p* > 0.05). In 2022, the onset of growth in the pure and mixed stands was estimated on DOY 70 ± 9 and 67 ± 2, respectively, with the end of growth occurring on DOY 351 ± 3, resulting in a growing season lasting 281 ± 5 days and 284 ± 3 days. In 2023, the onset of growth in pure and mixed stands occurred on DOY 67 ± 4, and the end of growth was estimated on DOY 351 ± 2, with a growing season lasting 284 ± 2 days (Table 2). However, the total xylem cell number and xylem growth rate in mixed stands were significantly higher than those in pure stands (Figure 2, *p* < 0.001). Based on the Gompertz equation, the average growth rate for pure and mixed stands was 0.10 cells day^−1^ and 0.22 cells day^−1^ in 2022 (Table 2). In 2023, the average growth rate was 0.13 cells day^−1^ and 0.16 cells day^−1^ in pure and mixed stands, respectively. In the mixed stands, the total xylem cell number reached 48 ± 3 in 2022 and 38 ± 3 in 2023. Similarly, in the pure stands, the total xylem cell number reached 25 ± 3 in 2022 and 28 ± 2 in 2023.

## 3. Materials and Methods

### 3.1. Study Site

The field experiment was carried out at the Experimental Center of Tropical Forestry (22.1138 N, 106.7800 E; 550 m a.s.l.), located in the south of Guangxi Zhuang Autonomous Region, China. The region has a typical subtropical monsoon climate, named Cwa according to the Köppen-Geiger classification [35], characterized by hot and humid summers and cool, dry winters. The mean annual precipitation is 1360 mm, with 78% of the total rainfall occurring during the wet season (May–September). The mean annual temperature is 21.7 °C, with the maximum monthly mean temperature reaching 27.9 °C in July and the minimum monthly mean temperature dropping to 13.6 °C in January. The soil is loamy, with a pH ranging from 3.8 to 4.8.

### 3.2. Experimental Design and Data Collection

Two plantation types with similar topographies, soil textures, and management practices were selected: a monoculture of 63-year-old Masson pine and an unevenly aged mixture of 63-year-old Masson pine with 39-year-old *C. hystrix* (Table 2). In 1959, Masson pine stands were established with an initial planting density of 2500 trees ha^−1^ in this area. One-year-old seedlings of *C. hystrix* were planted in 1983 after a clear cutting of the previous Masson pine stands, with a spacing of 2500 trees ha^−1^, thus reaching a proportion of 1:1 between pine and *C. hystrix* [33]. The two stands experienced similar management practices and were subjected to thinning in 1993, 1998, and 2009 to remove suppressed trees. After thinning, the stand densities across all the stands were adjusted to 1500–1650 trees ha^–1^, 1050–1200 trees ha^–1^, and 525–625 trees ha^–1^, respectively [33,36].

**Figure 4:**
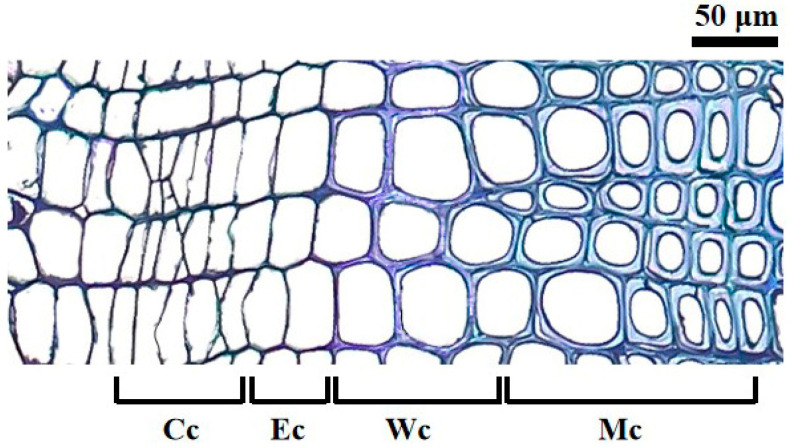
Cambium and different phases of xylem development of Masson pine: Cc cambium cells, Ec enlarging cells, Wc wall-thickening cells, Mc mature cells.

Nine healthy trees with straight stems were selected randomly from each stand. Microcores (2 mm in thickness and 1.5 cm in length) containing the last three to five years of annual rings were sampled from January to December 2022 and 2023. Microcores were extracted biweekly along the stem at a height of 1.0–1.3 m using a trephor [37], fixed in microtubes containing 50% ethanol, and stored at 4 °C. After being dehydrated subsequently in ethanol and D-limonene, the samples were embedded in paraffin, and 8 µm thick sections were cut with a rotary microtome (Leica RM2235, Leica Biosystems Nussloch GmbH, Nußloch, Germany). The developing xylem cells were observed using a Leica microscope (Leica Biosystems Nussloch GmbH, Nußloch, Germany) at a magnification of 100× after staining with 0.06% cresyl violet for 1 min [38]. The number of cells, including cambial cells, enlarging cells, wall-thickening cells, and mature cells, were recorded in three random files according to the strict identification standard (Figure 4). Enlarging cells are defined as being at least twice the size of the cambium cells. Cresyl violet acetate reacts with lignin and induces shining of the secondary walls under polarized light, making the wall-thickening cells appear purple. Xylem cells were considered mature when turning completely blue [39]. The total number of xylem cells were calculated as a sum of enlarging, wall-thickening, and mature cells.

### 3.3. Parameters of Simulated Xylem Formation Dynamics

The Gompertz function was used to simulate the total number of xylem cells for each tree [40]. The Gompertz function is defined as:

$$y=Aexp\left[{-e}^{\left(\beta -\kappa t\right)}\right]$$

where y represents the total number of xylem cells produced at time t, t is the time expressed as the day of the year (DOY), A is the upper asymptote of the total xylem cell production, β is the *x*-axis placement parameter, and κ is the changing rate parameter. We identified the onset of the growing season as the DOY corresponding to the appearance of the first enlargement cell, and the end of the growing season as the DOY when no enlargement cell was present in the tree ring [39]. The duration between the onset and end of the growing season was defined as the growing season length. The average growth rate during the whole growing season was the ratio between the number of xylem cells and the length of the growing season. To identify the differences between treatments, we conducted one-way ANOVA, with treatment as the independent variable and year as the replication.

## 4. Discussion

### 4.1. Enhanced Xylem Cell Production in Mixed Stands

Our results demonstrated that the xylem cell production of Masson pine in mixed stands was higher than that in pure stands, which is consistent with previous studies [6]. Bosela (2019) [26] measured the tree-ring width of 334 beech and 280 fir trees from 75 inventory plots in Slovakia and found that the higher diversity (expressed by Shannon’s index) was related to a greater basal area increment. In our study, the difference in the growth and physiological characteristics between *C. hystrix* and Masson pine led to overyielding by reducing competition [7,8]. Also, the height stratification of these two species and complementary crown architectures promoted higher stand-level light interception. As a light-dependent species, Masson pine in mixed stands has a greater height than *C. hystrix.* Thus, the absorption of light was not affected by the mixed stand structure, which could lead to the synthesis and allocation of more carbohydrates for stem growth [41]. Furthermore, the interspecific interaction of *C. hystrix* and Masson pine could explain the overyielding of mixed stands. *C. hystrix* increased the content of litter amount and nutrient flux in the soil surface layer, leading to faster litter decomposition rates and fine root turnover, which may contribute to enhanced soil nutrient availability [42] for Masson pine.

The effects of mixed stands diverge in the literature, with positive, negative, and null effects, depending on the species composition, site condition, or age of trees [43]. Multispecies plantations with opposite functional traits (leaf morphology, leaf life span, and nitrogen acquisition strategy) may produce higher complementary effects through niche partitioning [6]. Masson pine (a needle-leafed species) and *C. hystrix* (a broad-leafed species) exhibit distinct light interception strategies, characterized by spatial (leaf morphology and canopy position) and temporal (leaf phenology) variations, which promote complementary light utilization, optimize canopy carbon assimilation, and support arboreal growth. Moreover, studies have demonstrated that the mixed effect could occur only after canopy closure, which generally happens after the seventh year [27,44]. In early developmental stages, high structural heterogeneity may lead to lower productivity. In later developmental stages, however, stand structural heterogeneity had a positive effect on productivity [45]. In our study, after 39 years of mixed management, at the mature phase of Masson pine, we could still observe the positive effect of the mixed stands.

### 4.2. Increased Xylem Growth Rate in Mixed Stands

We found that there was no significant difference in the xylem phenology of Masson pine, i.e., the onset, cessation, and the growing season length between pure and mixed stands. However, the growth rate in mixed stands was significantly higher. This suggests that xylem cell production increases in mixed stands were due to the higher growth rate rather than longer growing season length. The growing season length and growth rate jointly determined the xylem cell production. In cold climates, the duration of xylogenesis was 14 days longer, which corresponded to a massive increase in cell production (33 cells) in Northern Hemisphere conifers [14]. In recent years, more studies have confirmed the decisive role of growth rate on xylem cell production [46,47]. Qian (2023) revealed that 86% of the xylem growth of Korean pine (*Pinus koraiensis*) was attributed to the growth rate and 14% to its duration in northeastern China [48]. Moreover, in arid and semi-arid climates, 69.9% of the variability in xylem cell production was attributable to the growth rate for the lower tree line at the Tibetan sites [49]. This indicates that the radial growth strategy of trees varies with climatic conditions. Given that the Masson pine in both pure and mixed stands were grown under the same climatic conditions, the difference in xylem phenology could be considered marginal. Thus, the mixing effect mainly leads to overproduction by increasing the xylem growth rate.

The higher growth rate in mixed stands may be associated with water availability and nonstructural carbohydrate (NSC) supply levels [19,28]. Cambium cells break dormancy and begin to divide after spring temperature and water conditions reach a certain threshold. Subsequently, carbohydrates combined with water molecules are allocated to xylem cells, generating water turgor pressure during cell expansion. This is followed by the production of polysaccharides for cell wall thickening. The xylem growth rate depends on the concentration of NSC in cambium, mobile sugars (e.g., starch, soluble sugar), and sugar alcohols (e.g., pinitol) to regulate cell osmotic pressure at the expense of growth when there is a water deficit [19]. The higher xylem growth in mixed stands could be related to a more favorable growth environment for Masson pine by enhancement of the local microclimate, mainly soil nutrient and water availability, which have increased the xylem growth rate and cell production. Interplanting broad-leafed tree species into Masson pine pure stands can significantly increase the amount of forest litter and litter components and enhance the content of soil organic matter [50] and soil water storage in the surface layer (0–20 cm) of the forest, providing a more adequate nutrient and water environment for the growth of Masson pine. On the other hand, the fine roots in mixed forests showed higher specific root lengths and root nitrogen concentrations, as well as lower diameters and root tissue densities compared to mono–specific stands [42,51,52]. These enhancements greatly improved the absorption and utilization of root resources (water and nutrients), which in turn promoted the aboveground growth (photosynthesis and wood formation) of the trees [26]. In addition, studies have shown that the main carbon source for the growth of conifer trees comes from NSC stored in leaves rather than trunks and branches [53], and its distribution pattern is affected by environmental factors (light, water, CO_2_ concentration, nutrients, etc.). When light is insufficient and encounters drought stress in the environment, the soluble sugar in the plant will be preferentially converted into starch for storage, thereby reducing the growth rate to survive the adverse conditions. Therefore, mixed stands have a more stable stand structure than the pure stands. Under the same climatic conditions, they exhibit stronger resistance to environmental stresses, and NSC is preferentially supplied to tree growth [54], thereby increasing the growth rate of tree xylem.

### 4.3. Application in Forest Management

Subtropical forests have been proven to show significant potential as a major regional carbon sink [55]. However, due to low resilience, they are vulnerable to extreme climate events caused by global warming, such as severe drought [56]. Through the process of wood formation, forests capture a substantial amount of anthropogenic carbon emissions and serve as one of the main terrestrial carbon reservoirs [57]. However, the prevalence and extent to which carbon assimilation (source) or xylem formation (sink) mediate wood production are fundamentally important and remain elusive [58]. While forests may increase carbon uptake due to rising temperatures and atmospheric CO_2_ concentration, increasing water stress has limited wood formation over the same period, reducing the annual increase in carbon in woody biomass [59]. Tree productivity and forest carbon sequestration will only increase when soil moisture is sufficient to maintain wood formation [60]. Our study showed that mixing age cohorts of the coniferous species Masson pine and the broad-leafed species *C. hystrix* could significantly increase the xylem growth rate, thereby amplifying the xylem cell production of Masson pine and ultimately enhancing the potential of forest carbon sinks. Our micro-core sampling and high resolution of the intra-annual xylem formation can facilitate the precise quantification of tree growth, providing novel insights for assessing the carbon sequestration potential of forests and furnishing a robust framework for evaluating forest management policies.

Species richness has been strongly associated with increased stand-level productivity [5]. Inappropriate species mixtures in forests may result in lower productivity compared to the corresponding monocultures [24]. We found that Masson pine in the pure stands was taller than in the mixed stands, indicating that Masson pine adopted different carbon allocation strategies in pure and mixed stands. Due to intense interspecific competition in pure stands, Masson pine allocates some carbohydrates to height growth to secure adequate light resources, reducing the proportion allocated to stem growth. However, in mixed stands, Masson pine is taller than *C. hystrix.* Thus, the growth is not limited by light resources, which could facilitate the allocation of more carbohydrates for radial growth. Consequently, the xylem cell production in the mixed stands was significantly higher than in the pure stands. In addition, the DBH of Masson pine in a mixed forest was smaller than that in a pure forest, which indicated that the un-aged mixing for 39 years had a negative effect on the growth of Masson pine. However, by analyzing the intra-annual growth of xylem, we found that the growth rate of Masson pine in a mixed forest exceeded those in a pure forest, which indicated that the mixed effect had a time lag, and there was no obvious positive effect in the early stage of stand establishment [45]. With the extension of stand management time, the mixed effect was more significant [27].

Furthermore, selecting the appropriate mix of tree species is crucial for increasing productivity. In addition, plant diversity promotes community productivity through niche partitioning among species, which allows for efficient utilization of resources (light, nutrition, and water) [61]. Mixed planting of nitrogen-fixing and non-nitrogen-fixing species can improve soil nitrogen absorption efficiency and enhance stand productivity [62]. Differences in shade tolerance among species affect competition for light and lead to variation in horizontal and vertical canopy structures, thereby improving light use efficiency [22]. Our study demonstrated that, according to the different growth dynamics of xylem in various tree species throughout the year, the differentiation of ecological niches can be carried out to better profit from the available resources. The difference between species is an important factor affecting the radial growth of trees. Evergreen species were able to initiate wood formation during the short rainy season in southeastern Ethiopia, whereas growth in the deciduous broadleafed species started during long rainy seasons [11]. Thus, we suggest that planting trees with different xylem phenology could maximize resource utilization to enhance the growth and resilience of forests.

## 5. Conclusions

Transforming pure conifer forests into mixed stands is vital for sustainable development in subtropical forests of southern China. After monitoring the xylem formation dynamics of Masson pine in pure and mixed stands for two consecutive years (2022–2023), we found a higher xylem production in the latter, which indicated a positive effect of mixed stands on the growth rate of pine. Interspecific complementarity and interaction promoted higher resource utilization efficiency of Masson pine, which in turn accelerated xylem cell growth during the growing season. Furthermore, we found that the mixed effect did not affect the xylem phenology of pine. There was no significant difference in the onset, ending, or duration of the growing season between mixed and pure stands. Xylem phenology is more influenced by macro- and meso-climates rather than microenvironmental conditions. Our results demonstrate that monitoring the annual xylem growth dynamics can be an important tool to evaluate the effect of species composition and reveal the mechanisms to promote tree growth behind the mixed effect. Different tree species adopt different growth strategies, and quantifying the xylem cell production is of great significance for Masson pine to choose appropriate mixed tree species and support more efficient and sustainable forest management strategies.

## Figures and Tables

**Table 1:** Analysis of variance (ANOVA) results of the key parameters of xylem formation between pure and mixed stands of Masson pine.

Year	Xylem Growth Parameters	Pure Stands	Mixed Stands
2022	Onset (DOY)	70 ± 9	67 ± 2
	End (DOY)	351 ± 3	351 ± 3
	Growing season length (days)	281 ± 5	284 ± 3
	Total cell number (cells)	25 ± 3	48 ± 3
	T-max (DOY)	168 ± 8	188 ± 5
	Average growth rate (cells day^−1^)	0.10 ± 0.02	0.22 ± 0.015
2023	Onset (DOY)	67 ± 4	67 ± 4
	End (DOY)	351 ± 2	351 ± 2
	Growing season length (days)	284 ± 2	284 ± 2
	Total cell number (cells)	28 ± 2	38 ± 3
	T-max (DOY)	141 ± 4	155 ± 3
	Average growth rate (cells day^−1^)	0.13 ± 0.005	0.16 ± 0.004

**Table 2:** Characteristics of Masson pine in pure and mixed stands in the south of Guangxi Zhuang Autonomous Region of southern China.

Treatment	Species	Stem Density (Tree ha^−1^)	DBH (cm)	Height (m)	Canopy Coverage (%)
Pure stands	*Pinus massoniana*	358 (325−400)	45.4 ± 9.0	25.6 ± 3.2	77.3 (73.7–79.9)
Mixed stands	*Pinus massoniana*	242 (225−275)	41.1 ± 5.7	20.6 ± 3.3	83.5 (82.5–84.7)
	*Castanopsis hystrix*	325 (300−350)	22.1 ± 6.5	18.1 ± 2.2	

## Data Availability

The data that support the findings of this study are available from the corresponding authors upon reasonable request.
